# Comparison of Filtering Methods for Enhanced Reliability of a Train Axle Counter System

**DOI:** 10.3390/s20102754

**Published:** 2020-05-12

**Authors:** Damian Grzechca, Adam Szczeponik

**Affiliations:** 1 Department of Electronics, Electrical Engineering and Microelectronics, Silesian University of Technology, 44-100 Gliwice, Poland; Adam.Szczeponik@rail.bombardier.com; 2 Bombardier Transportation, 40-142 Katowice, Poland

**Keywords:** railway wheel detection, axle counter, signal processing, filtration

## Abstract

This paper presents signal filtering methods that can be effectively applied to train detection systems based on the axle counter systems that are currently in operation for train detection and provide information on the unoccupied status of railway tracks and turnouts. Signals from the wheel detectors contain noise, may be impulsive and time-varying, which means that even for the same train, the signals from the following wheels may be different. A problem appears when already homologated hardware (axle counter system) is working in a harsh environment, exposed to disturbances whose parameters significantly exceed standard thresholds. Despite this, the system must continue to provide reliable information. The authors present research on the application of such filters as median, Savitzkey-Golay, and moving average which can be implemented in the equipment currently in use under specific constraints (e.g., limited computational resources). The research results show that appropriately adjusted filters, for example, in terms of type and window size, increase the signal quality and thereby provide reliable information about passing trains, as well as enhance the availability and safety of the axle counter system performance.

## 1. Introduction

Train control systems are crucial components to ensure a safe and efficient operation of railways. Researchers try to enhance the reliability of transportation in a variety of manners, namely by constructing a decision support system for railway traffic management [1], or by developing suitable condition monitoring techniques to ensure early, preventive diagnostics for railway systems [2,3]. In order to make transportation safe, rail inspections should also be done properly. An efficient crack (broken rail) detection system is proposed by Kumar et al. [4] for better diagnostics and inspection. Research is also carried out in the direction of using obstacle detections systems (study for image processing presented by Yao and colleagues [5]). Nowadays, such systems are commercially used for monitoring lines (e.g., distributed acoustic sensing based on fiber optic technology) and level crossings (e.g., radars).

A study on the need for train detection is presented by Palmer [6]. Authors highlight their crucial role for traffic management and safety. In the past traditional track circuit was used as a system for detecting non-occupancy of sections; in recent year they have been successively replaced by axle counters [6].

An axle counter, which is used for train detection, has currently been one of the most important railway signaling systems, that should provide reliable and safe information for train control purposes. In order to fulfill this task, specific sensors (detectors) are used to confirm the integrity of a train and to indicate if a track section is unoccupied [7]. Other sensors may indicate independent measurements of speed, for example. Nowadays, axle counters are the most popular type of train detection systems and a critical part of the rail signaling world. Axle counters provide vital information about non-occupancy of tracks and turnouts for level crossing systems, interlocking systems and line block systems [8].

Proper and safe operation of this system is mandatory for managing traffic on railway lines. A typical axle counter system consists of two main components: a wheel detector and an evaluation unit. The functions of these two components differ in various system architectures. In one architecture the detector is used only for detecting the presence of a wheel of the rolling stock [9], while in the other architecture a wheel detector is responsible for detecting and counting the wheels passing the point where it is installed. Yasukawa et al. [10] designed an optimization of the axle counter through large-scale magnetic field computations. They estimated the signaling level by applying infinite edge elements to an extensive air domain. Futsuhara and Mukaidono [9] indicated some disadvantages of the wheel detectors including long-term variations in the mechanical tuning or the generation of an unbalanced signal due to the occasional presence of metal objects (empty cans, etc.); when the wheel is passing, the detector can either produce a positive detection signal or a negative detection signal. The positive and negative detection signals have no relation to each other. Moreover, the signal from the wheel detector contains unwanted components and noise [11,12,13] that should be removed safely. There are well known approaches for smoothing (filtering) various signals [14]. Safety-related systems require standards [15] but there is also space in supporting systems for fault diagnosis [2,16,17,18] and predictive maintenance [19] like fuzzy expert systems [20], neural networks [21], and also a filtering and smoothing algorithm [14]. However, the utilization of non-deterministic methods in a safety-related system (SIL-4) would be difficult due to uncertainty of the outcomes.

A typical, inductive wheel detector head contains separate coils and produces two signals, each of them in a separate channel. Thus, the evaluation unit can be responsible for analyzing the signal received from the detector, counting the wheels and calculating the balance of the wheels (axles) counted in sections or just calculating the balance when the number of wheels is provided directly by a wheel detector (which is called an axle counter unit in this architecture). Nevertheless, there is always a component of the system which is responsible for counting the wheels passing the place where the detector is installed. The function described above must be performed in compliance with the highest safety integrity level (SIL-4 according to the CENELEC standard) [15,22]. For such functions, the tolerable hazard rate (THR) (i.e., probability of an incorrect, hazardous operation) shall be less than 10^−8^. It is done and satisfied by the company solution and cannot be published. The wheel detector is installed directly on a rail. Typically, this inductive sensor in a non-contact manner detects the presence of a wheel of a rail vehicle. The occurrence of the wheel, made from ferromagnetic material in the area of the detector operation, changes the parameters of the electromagnetic circuit and as a result influences the output signal of the sensor. Based on the signal analysis, the presence and passage of the wheel is detected. Various designs of the coil unit allow for the construction of detectors which operate thanks to a change of different parameters of this electromagnetic circuit, which results in amplitude, phase, or frequency modulation of an output signal. All of the listed methods have both advantages and disadvantages. Some of them are highly immune to electromagnetic disturbances (caused by rolling stock or other railway systems [23]) and at the same time are highly sensitive to the position of a sensor [9]. Zamani and Mirabadi [24] presented a method for searching for the best orientation of coils to achieve the highest sensitivity of a sensor and thus obtaining the best relation between the signal and noise. Another way of improvement is to search for methods other than inductive technologies for train detection. Brockmann and colleagues [25] discussed utilizing the acoustic wave radiated by the train and its analysis in the frequency domain. Other research focused on using fiber optics as a source of information for a train’s presence. One of the most advanced research works concerns Fiber Bragg Grating (FBG) as a sensor of the tensions in rails generated by the train, which can be utilized for axle counting [26] or for rail diagnostic purposes [27].

The goal of the analysis, presented in the paper, is to find a method to increase the quality and improve the performance of the existing systems. Thus, the hardware platform is considered fixed because of the costs. There are already thousands of detectors installed around the world, for which software upgrade is the only acceptable option for improvement. Any hardware modification (i.e., a replacement of the existing hardware with the new one) is not feasible for financial reasons. Another reason is that such a type of system is subject to validation and a homologation process. A high number of tests (e.g., environmental tests, electromagnetic compatibility tests) and analyses need to be repeated when a hardware platform is changed. Any software modification requires re-testing as well, however test scope can be limited (e.g., failure mode and critical effect analysis (FMCEA), temperature tests, vibration tests, and electromagnetic compatibility (EMC) tests in the scope of radiated and conducted emissions can be skipped when hardware platform and safety assurance methods are not changed). Among various methods of signal processing, filtration is the fundamental one and thus presented in this paper. It needs to be noted however, that the amount of time available for signal processing is highly limited and computational resources are fixed.

The article filtration methods [28,29] are intended to be applied in the double head detector whose operation is based on the amplitude modulation of the signal caused by a passing wheel. As described before, hardware construction is considered fixed and cannot be changed. All implemented methods have to fit to the existing hardware platform with all of its constraints (in this paper the limitation is: 16 kHz sample rate per channel, two channels for processing, fixed point operation, 30 µs of available time for signal processing/3500 of atomic operation within available time).

**Figure 1:**
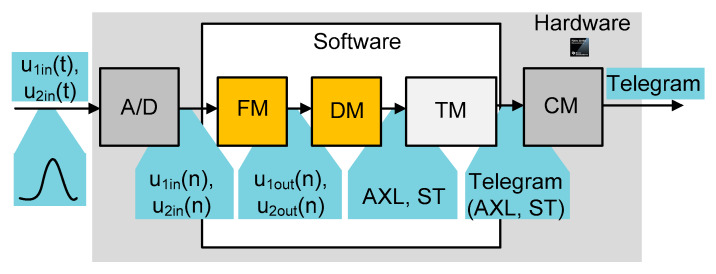
Data flow in the software signal processing path in wheel detector. A/D: analog to digital converter; FM: filtration module; DM: decision module; TM: transmission module; CM: communication module; Telegram: a digital telegram that is sent via a transmission link; AXL: the number of counted axles; ST: status of operation (error codes). The signals between the following blocks are as follows: u_1in_(t): analog, input signal, channel 1; u_2in_(t): analog input signal, channel 2; u_1in_(n): digital, input signal, channel 1; u_2in_(n): digital, input signal, channel 2; u_1out_(n): digital, filter output signal, channel 1; u_2out_(n): digital, filter output signal, channel 2.

The input signal, which comes from the wheel detector heads is filtered and demodulated in the hardware part of the detector. Software processing starts just after analog to digital conversion of a demodulated signal (envelope) and ends on the output of a decision module, which provides data about the number of counted axles (wheels) and status, containing information about the state of the detector operation and abnormalities of the input signal. The path of the signal processing is presented in Figure 1.

It has to be emphasized that the main goal of the research is application of a different filtration method, which can be implemented inside the “Filters Module”.

## 2. Wheel Detector Signals

Data (signals) used for testing purposes (i.e., test files containing recorded real signals u_1in_, u_2in_ that are used as input signals for experimental purposes) were collected from the existing installations of Bombardier’s axle counter systems in commercial operation. The data was collected manually (i.e., measured during site visits or recorded automatically by autonomous recorders like signal logging devices) and installed in the wheel detectors in various locations all over the world. Only the files containing signals which caused problems with axle counting in the existing axle counter system (i.e., disturbed the operation of the wheel detectors) were chosen for the experiments (in the existing system the wheel detectors report errors as they are not able to count axles based on built-in criteria).

The number of axles recorded in each test file is known: directly, for the site measurements performed for certain trains, and generally for all train passages where the type of train is known or indirectly as the number of axles has been identified during the test files analysis performed by Bombardier’s signaling specialists (based on review of the recorded signals in relation to information logged in the axle counter evaluation unit black boxes and interlocking system logs, if needed). This information allows to define the criteria for test results.

The input signals u_1in_(t) and u_2in_(t) are low frequency signals measured on the input of a microprocessor of the trackside electronic unit of the wheel detector. These signals are the outcome of evaluation of wheel detector head signals (sinus, ca. 50 kHz), passing through the analog part of the wheel detector. This path consists of an instrumental amplifier with band limited to 100 kHz, an anti-aliasing filter with band limited to 60 kHz, a band-pass filter with band limited to 2 kHz around the center frequency (in order to protect end low pass switching capacity filter), and a synchronic demodulation unit with a tenth order low pass (switching capacity) filter which limits the band to 300 Hz. This path protects the recorder against aliasing phenomena. In the measured signal there is no higher component present than 300 Hz. The sampling rate of the autonomous recorder used for capturing signals was 3 ksps/channel.

### Tests Signals

For experimental purposes, the authors decided to choose tests signals for the following scenarios: (1) signal of train passages in one direction, without stops and returns, strongly disturbed, and (2) signal without train passages, containing only noise/disturbances. In order to evaluate filtration methods, case (1) contains a known number of axles and case (2) contains no axles. The data processing can be performed on signals u_1in_(*n*) and u_2in_(*n*) because the analog to digital converter (A/D) module is fixed and cannot be replaced (homologated hardware platform).

**Figure 2:**
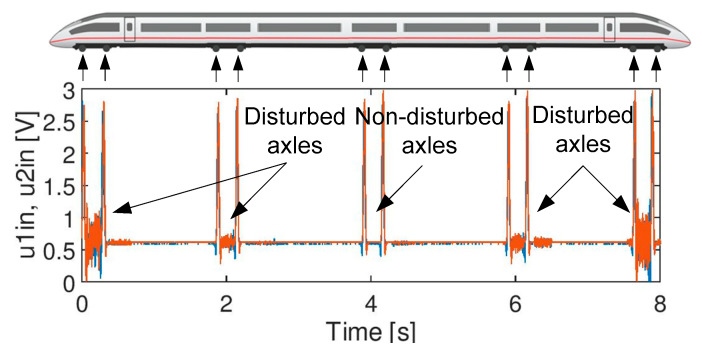
Signal quality for exemplary train passage.

Analysis of the genesis and nature of the disturbances overlapped on the axle signal, chosen for experiments is beyond the scope of the research presented in the paper. However, only the signals influenced by electromagnetic disturbances were considered. The source of some are already known, while others are not yet known. Single spikes are mainly caused by arc on the catenary, which may occur during rising or falling of the pantograph. The shape of a signal during the rising differs from the one which occurs during the falling as the nature of discharge is also different. Such spikes can also be generated during the train passage through a neutral section with full power. Regular spikes have been recorded only in a single application and their source is still unknown. It is suspected that they are generated by an onboard system which can either be in testing mode or is not working correctly because it is faulty. It is already known that vehicles in operation in this location are not equipped with the filters on the power feeding system. Continuous disturbances are caused by the return current flowing through the rails and are produced by traction systems of the vehicles. Some of them (of a similar shape) are caused by the ice accumulating on the catenary (arc and sparks during the train passage). Disturbances existing only in the middle of the bogie are mainly caused by radiated disturbances from the traction units (schematically shown in Figure 2).

Other factors which have an impact on the wheel detectors signal, like extensive sideways movement of a wheel (wheel flange is not located directly over the detector head), small wheels (of small diameter, poor flange, spike wheels, wear and tear), or additional metal equipment mounted into the wheelsets (in the metal free zone) were not taken into consideration for the research on filters. Such signals should be dealt with through an appropriate decision algorithm design.

The electromagnetic compatibility of axle counter systems with rolling stock and infrastructure is presented by Dercosi Persichini and colleagues [30]. Based on the research and work of normative groups a couple of standards addressing this issue have been released (e.g., EN 50121, CLC/TS 50238, EN 50617). Although this is also regulated by European Union (EU) Directives (e.g., 2008/57/EC), improvement and better solutions are still sought. Firstly, this is because not all of the systems in operation on the railway network meet the currently valid requirements, and secondly, the condition of infrastructure and vehicles may vary depending on location and changes over time, thereby generating availability issues.

**Figure 3:**
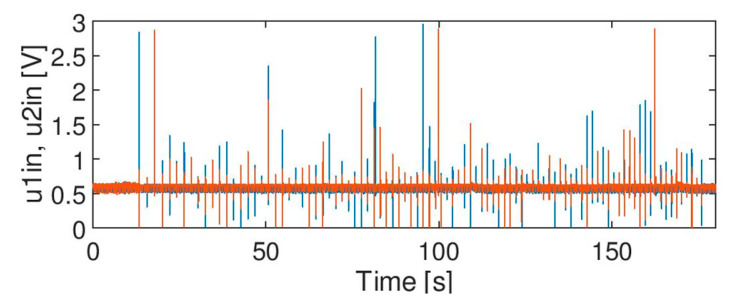
Signal A: peaks of high values for no axle case.

**Figure 4:**
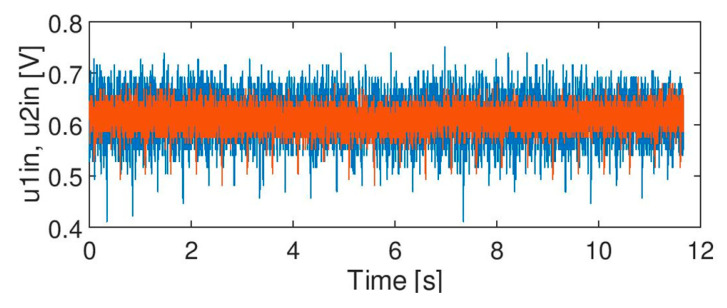
Noisy signal, no axle (original one, before multiplication).

**Figure 5:**
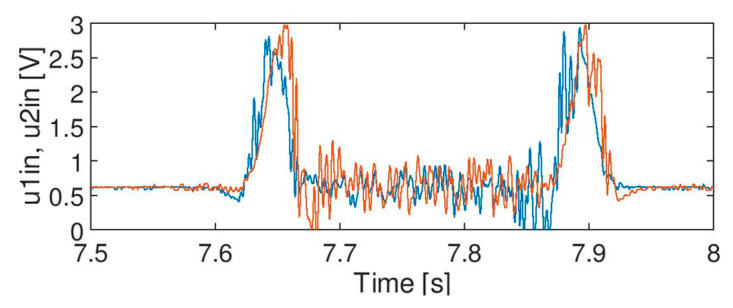
Signal quality during train passage, an example.

The following test signals were used in the experiments:

- Signal A (Figure 3): containing spikes/peaks of high values over the time slot of acquisition under no train condition; no axles. The nature of the phenomenon is unknown.
- Signal B (Figure 4): noise of high variation: no axles. Apart from noise, it also contains life test impulses for experiment purposes, to create the worst case scenario and thus a robust filter, signal B is multiplied by factor 2.
- Signal C (Figure 5): signals from two heads of the wheel detectors with noise. It is a signal indicating a train passage for further data processing software.

The aforementioned signals are categorized and acquired from various locations. A data processing software which relies on threshold values and duration (time) was designed but does not allow for no error solutions for signals A, B and C.

**Figure 6:**
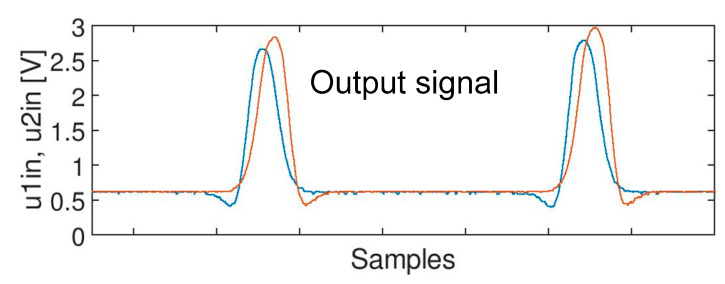
Desired output but filtered signal for train passage.

The goal of the research was to eliminate unwanted components from the input signals A, B and C with minimum impact on the main information carried by the signal (as shown in Figure 5). There should be no impact (or it should be as low as possible) on the shape of the axle signal (e.g., the amplitude should not be decreased, and duration should not be changed). An example of the desired result is shown in Figure 6.

## 3. Data Processing

**Figure 7:**
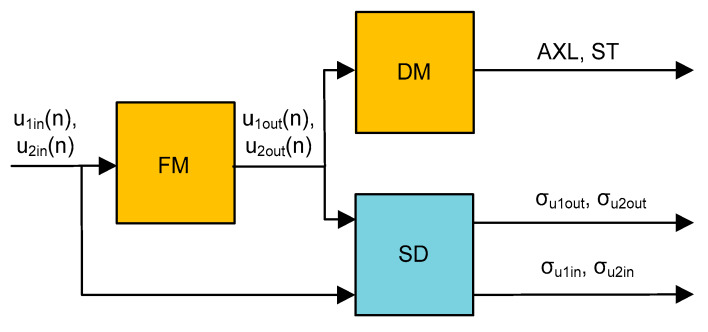
Data processing diagram.

Since the hardware was designed a number of years ago, the hardware constraints (microcontroller, memory, clock, etc.) must be considered. To assess the effectiveness of the filtration method a selected test signal (described in Section 2) was fed to the input of a filtration module (FM), shown in Figure 7. Two additional modules, namely, a decision module (DM) and a statistic description (SD), were introduced to evaluate the results. The idea of the signal flow takes into account the applied filter (FM output signal) which is processed in the decision module (details are described further in Section 3.1). The DM is responsible for the calculation of the number of axles (AXL) and the status of operation (ST) (i.e., error code). The AXL is compared with the known real one (expected value). An error code value equal to 0 is considered as no error. The SD module is described in Section 3.3. In general, however, it mainly compares the quality of the signal based on the standard deviation values (*σ*) of the raw input signal and the filtered one.

### 3.1. Decision Module

**Figure 8:**
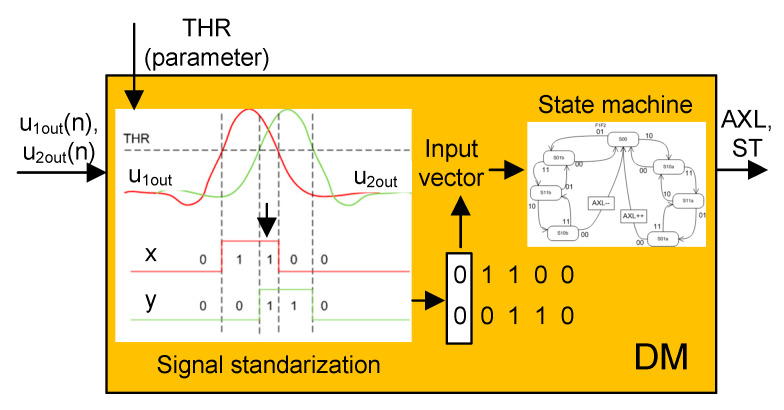
Decision module (DM).

The decision module (DM) is responsible for providing information about the number of axles (AXL) and the status of operation (ST). In other words, it assesses the filtration effectiveness. For the purpose of acquiring the output information, a state machine was designed. It enables detecting a passage of a wheel as a sequence of two signals that are received from the wheel detector heads. The signals from both heads (shifted by about 20 cm) are then demodulated, filtered, and standardized (digitized) to 0/1 values based on the adjustable threshold level (THR). The THR must be adjustable because it depends on the amplitude which is modulated by the passing wheels (issues are described in Figure 3, Figure 4 and Figure 5). Raw signals go through the synchronic demodulation unit (hardware implemented phase sensitive demodulation method) and can be filtered in the module (FM). The output signal is digitized based on the configured threshold level (three values are applied: 1.6 V, 1.2 V, 0.95 V). Thus, the output vector contains the series of 0/1 samples created by both wheel detector heads. Each pair of samples is processed in the state machine and the number of axles counted, or if an improper sequence is detected, an error is indicated as shown in Figure 8.

### 3.2. State Machine

**Figure 9:**
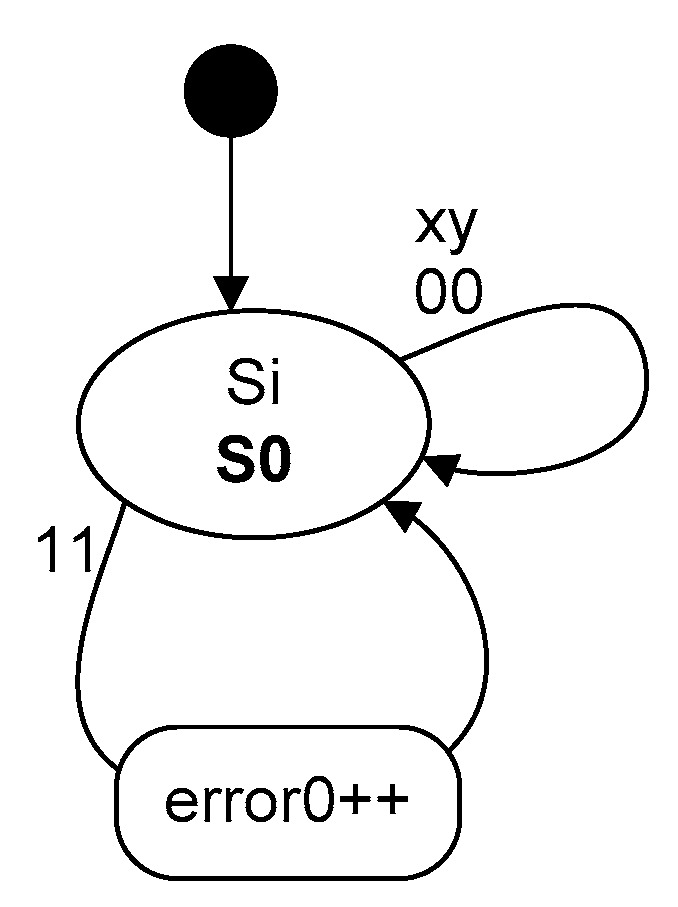
State machine start-up.

As mentioned, the state machine (SM) was designed for digitized signals which come from a wheel detector head. The signals from the heads are denoted as x and y, respectively. Thus, xy creates an input vector for a state machine (e.g., xy = 00, 01, 10, 11). The SM can be in one of the seven states, (i.e., Si for i = 0…6.) The following parameters are considered in respect of the state machine operations:

- *axles*: number of axles counted after processing of the input vector xy
- *errorX*: number of occurrences of incorrect transitions (accumulated). *X* is related to the type of sequence detected (for start-up: error0: 00 → 11, Figure 9)

**Figure 10:**
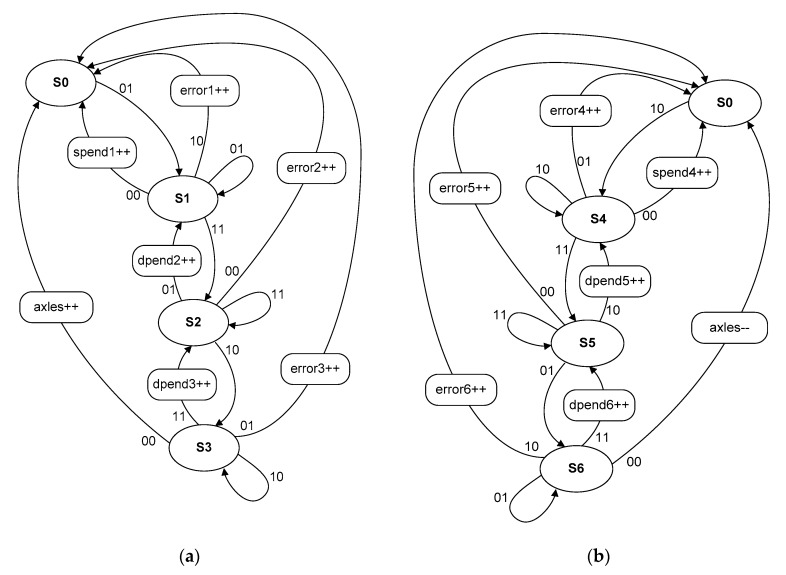
State machine: positive direction/move forward (**a**), negative direction/move backward (**b**).

The operation state-machine (Figure 10):

For right branch (Figure 10a): error1: 01 → 10; error2: 11 → 00; error3: 10 → 01;

For left branch (Figure 10b): error4: 10 → 01; error5: 11 → 00; error6: 01 → 10.

In case of a clear, undisturbed signal the value of *errorX* shall be 0.

- *spendX*: shallow pendulum detected. *X* is related to the type of transition. It is a normal situation in real traffic, especially in the case of shunting movements. For the test purpose it is assumed that there is no such sequence of signals stored in the test files. With respect to clear passages the value of *spend*X parameter shall be 0. A shallow pendulum is detected if and only if solely half of a detector head is occupied by the wheel (one channel of the head is activated).
- *dpendX:* deep pendulum detected. *X* is related to the type of transition. It is a normal situation in real traffic, especially in the case of shunting movements. For the test purpose it is assumed that there is no such sequence of signals in the test files. With respect to the clear passages the value of *dpendX* parameter shall be 0. Deep pendulum is detected when the whole detector head is occupied by the wheel (both channels of the head are activated).

For assessment purposes, the following description can be seen in the diagrams presented in Figure 9 and Figure 10: 

S0 ÷ S6—state of the state machine. For positive direction: spend1, dpend2, dpend3—counter for returns, error1 ÷ error3—counters for errors, input vector xy sequence: 00, 01, 10, 11.

Positive count (a) transitions (without returns) are:

S0 → S1…S1→ S2…S2 → S3…S3 → S0

00 → 01…01 → 11…11 → 10…10 → 00

For negative count direction (move backward): spend4, dpend5, dpend6—counters for returns, error4 ÷ error6—counters for errors, input vector xy sequence: 00, 01, 10, 11. 

Negative count (b) transitions (without returns):

S0 → S4…S4→ S5…S5 → S6…S6 → S0

00 → 10…10 → 11…11 → 01…01 → 00

Once the designed state machine is certified it cannot be changed because it produces a validated outcome. This is one of the reasons that the safety systems (certified once) are very difficult to revise. Therefore, under a validated solution another one can be implemented parallelly (i.e., the output values (axle detection) can be indicated by two or more algorithms).

The simplified designed state machine was utilized for the experiment. This is a solution which is not resistant to noise and can be applied in a system which functions based on disturbance-free signals (hardware is more immune/robust and provides better input). Therefore, such a state machine was used for assessing the filtration effectiveness.

### 3.3. Statistic Description Module

Another criterion used for assessing the filtration method was a standard deviation Equation (1) calculated for the output signal in relation to the input one. This parameter was calculated for all the evaluated signals namely signal A (no train passage), signal B (no train passage), and signal C (train passage). Standard deviations of the signals are calculated separately for each of two channels according to Equation (1).

(1) $$\sigma =\sqrt{\frac{{\left(x_{1}-\bar{x}\right)}^{2}+{\left(x_{2}-\bar{x}\right)}^{2}+\ldots +{\left(x_{N}-\bar{x}\right)}^{2}}{N-1}}$$

A separate criterion that must be considered without doubt for the assessment of the filtration method is the implementation costs of the algorithm. In particular, such parameters, like the number of coefficients, number of multiply operations, and memory consumption are taken into consideration for comparison of all filters under analysis.

## 4. Filters under Analysis

The main goal of the analysis is to achieve the status when low demand computational resources for filtration are combined with high filtration effectiveness and no false axle counts. The most common approaches to smoothing the real signals with limited resources are presented in other studies (see [31,32,33,34]). The cost of the algorithm is crucial; for example, the outcome decision for fast train passage in specific hardware conditions (limited sampling frequency, microcontroller clock speed) must be taken within a short period of time and filtration cannot change the relationship of signals from both detector heads (neither delay nor amplitude can be unsynchronized). One can notice that the disturbances are located in the band and it is not possible to cut the noise/disturbances out by the high order low-pass filter without affecting the core information. In such a case, the following filtration methods were considered [31]:

### 4.1. Moving Average Arithmetic Filter (MAA) 

It is one of the most popular filters in the digital processing world [28], mainly due to its simple implementation. It has low pass amplitude characteristics.

(2) $$y_{i}=\frac{1}{N_{w}}\overset{N-1}{\underset{j=0}{\sum }}x_{i-j}$$

*N_w_*—buffer size (window size).

The output sample is calculated as an arithmetic average of the input signal stored in an N-size buffer. For the causal filter, the sum is calculated for input samples before the output point.

### 4.2. Moving Average Arithmetic Square Filter (MAS)

It is a modification of the standard moving average filter, by rising each sample to the power of 2.

(3) $$y_{i}=\sqrt{\frac{x_{i}^{2}+x_{i-1}^{2}+\ldots +x_{i-N_{w}-1}^{2}}{N_{w}}}=\sqrt{\frac{1}{N_{w}}\overset{N_{w}-1}{\underset{j=0}{\sum }}x_{i-j}^{2}}$$

The idea of this formula is to increase the signal-to-noise-ratio (SNR), calculated as a mean divided by the standard deviation in comparison to the normal moving average filter [28].

### 4.3. Moving Average Geometric Filter (MAG)

Moving average (geometric) is calculated as a root of the product of the *N_w_*-size input buffer.

(4) $$y_{i}=\sqrt[{N_{w}}]{x_{i}\cdot x_{i-1}\cdot \ldots \cdot x_{i-N_{w}-1}}=\sqrt[{N_{w}}]{\overset{N_{w}-1}{\underset{j=0}{\prod }}x_{i-j}}$$

### 4.4. Median Filter (MF)

The output is calculated as a value, from which half of the population of samples is higher and the rest is lower (Equation (5)). For a sorted buffer of input samples, see below:

(5) $$y_{i}=x_{\frac{N_{w}+1}{2}}\{\begin{array}{c} x_{\frac{N_{w}+1}{2}},\;if\;N_{w}\;is\;odd\\ \frac{1}{2}\cdot \left(x_{\frac{N_{w}}{2}}+x_{\frac{N_{w}}{2}+1}\right),\;if\;N_{w}\;is\;even \end{array}$$

### 4.5. Savitzky-Golay (SG)

The Savitzky-Golay filter [31,32,33] is a generalization of the moving average filter. During the calculation each sample is multiplied by coefficients, which are products of the polynomial (Equations (6) and (7)).

(6) $$y_{i}=\overset{N_{wR}}{\underset{j=-N_{wL}}{\sum }}C_{j}x_{i+j}$$

*C_j_*—polynomial coefficients.

For analysis purposes, *N_wR_* = 0 (causal filter), and therefore the formula can be rewritten as:

(7) $$y_{i}=\overset{N_{w}-1}{\underset{j=0}{\sum }}C_{j}x_{i-j}$$

## 5. Results

The aforementioned filters were implemented for the purpose of assessment and were tested individually. Signals A, B and C categorized in Section 2 are the filter inputs. The decision module (DM) can be set into one of three thresholds—0.95 V, 1.2 V, and 1.6 V—and analysis of all the cases was performed. The expected number of axles (AXL) is described in the tables below, but it is obvious that the DM cannot (should not) indicate axles for signals A and B. The results of analysis are presented in Table 1, Table 2 and Table 3 for signals A, B and C, respectively. The first column is a filter type together with window size (*N_w_*). For the Savitzky-Golay filter, apart from the buffer size, the degree of the polynomial is also given.

According to Table 1, a median filter (MF) with window size of 64 is most efficient for signal A since there is no false axle counting (number of counted axles = number of expected axles = 0; lack of incorrect transitions in the state machine ST1 = ST2 = 0 (although disturbance level is very high)). It therefore can be concluded that a median filter is the best solution for filtering impulse noise. Moreover, the best results are not related with the threshold level in the decision module (DM). Such a filter shall be considered for use, however further analysis of its impact on the axle signal is required (discussed in Section 5).

**Figure 11:**
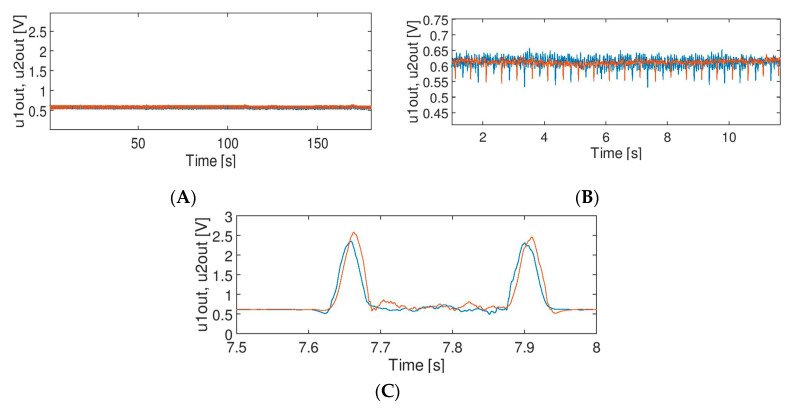
Filtration of the signals (**A**–**C**) (see Figure 3, Figure 4 and Figure 5).

Regarding Table 2, signal B has the best outcomes for moving average (arithmetic) filters of a window size of 64. In this case, there is one important thing which must be taken into account. Simple multiplication of original test signals (to increase the noise level) may lead to false results. As the dc component (offset) of the original signal is 0.6 V, it means that multiplication moves the signal to 1.2 V level (dc). It generates many false transitions in the DM for THR = 1.2 caused by the noise around the threshold level (for simplicity, no hysteresis for THR was implemented for signal standardization according to Figure 8). For THR = 0.95 many false transitions are caused by noise as well by life tests and negative impulses are visible in the signal. Since such effects may lead to the wrong conclusions, the test was repeated for signal B multiplied by factor 2 and shifted back to 0.6 V (being a nominal, static value of the signal). Results are presented in Table 2. It simulates higher noise level without changing the dc component, as it more probable in the real on-site conditions. With respect to such a modified test signal, the best results were achieved for moving average square filter (MAS). In this case a state machine (DM) is a poor quality factor for result assessment, as the whole output signal is kept below THR level and no false transitions are possible. The calculated minimum value of standard deviations proves that the moving average square (MAS) filter increases the signal to noise ratio compared to a typical moving average arithmetic (MAA) filter for the same window size. For signal C (train passage), the highest efficiency was obtained for two filters: moving average arithmetic (MAA) with a window size of 32 and Savitzky-Golay (SG) with a window size of 63 and third order polynomial. The number of counted axles is correct only with respect to these two filters—there was no false counting and no improper transitions—zero errors (ST1 = 0) and a minimal number of false returns (ST2 = 0). However, the best results for signal C were acquired for THR = 1.6 in the decision module. The filtration results that are indicated (in bold) in the tables are depicted in Figure 11.

Figure 11 shows the effect of filtration of the input signals A, B and C. The effect of filtration of signal A (Figure 3) by median filter MF (*N_w_* = 64) is presented in the Figure 11A. It shows that impulses were eliminated completely. This outcome is desired, but the impact of such a filter on the signal must be further verified (discussed in Section 5).

The effect of filtration of signal B (Figure 4) by moving average filter MMA (*N_w_* = 64) is presented in the Figure 11B. The noise level was significantly reduced, although Table 2 shows that MAS filter results were a bit better (i.e., a lower standard deviation of the output signal).

It needs to be noted, however, that the life test visible in Figure 11B (i.e., regular negative impulses discussed in more detail in Section 5) may affect the calculation of a standard deviation for noise with level comparable to the modulation depth. The real standard deviation of noise in the output signal is lower than the value presented in the table. It means that the actual quality of the signal after filtration may be better. The effect of filtration of signal C by moving average filter MAA (*N_w_* = 32) is shown in the Figure 11C. Similar effects of filtration were achieved for Savitzky-Golay filter SG (*N_w_* = 63, third order) and this will be further discussed.

## 6. Discussion

Smoothing filtration is a useful method of increasing the quality of signal which is affected by the disturbances within the band. The impact on the core information is however a disadvantage of such filtration. Incorrectly adjusted parameters of filtration (i.e., the length of the filter) may destroy/deform the signal or cut the signal out completely together with disturbances.

One can notice that impulse noise (signal A) is effectively removed by a median filter. However, if the signal comes from a fast-moving wheel, the impulses are short and the final solution (i.e., filter design) must take into account the maximum permitted speed of the train, for which the filter must work safely. Filter length (window size) must be selected carefully. For a real safety-related system, the worst case scenario assumes that the speed of the train is 450 km/h. The wheel detector head sensitivity area for a single channel is ca. 20 cm long. It leads to the duration of the impulse (worst case scenario) from a single wheel of 1.6 ms (for a threshold level THR = 0.95 V). Considering the fixed sampling rate of 16 kHz, it gives roughly 25 samples. In the case of window size of a filter below the doubled amount of collected samples, the impact to the signal is negligible. The output signal is just delayed, not deformed. For window size of 50, the output signal is significantly damped (for an even number of samples for the window size, the output is calculated as the average of adjacent samples) and disappears for a size of 51.

Hence, the median filter can have a significant and sudden impact on the signal. Its parameters, such as window size (buffer length), shall be selected in relation to the maximum speed of a wheel (train) at which a detector must operate reliably. A wheel detector is a safety-related system and it must consider a safety margin (at least 100% margin is usually recommended) to cover deviations resulting from various wheel construction, rail profiles, and way of movement, as all these mentioned factors influence the shape and thus the duration of a signal for a specific speed of a train.

**Figure 12:**
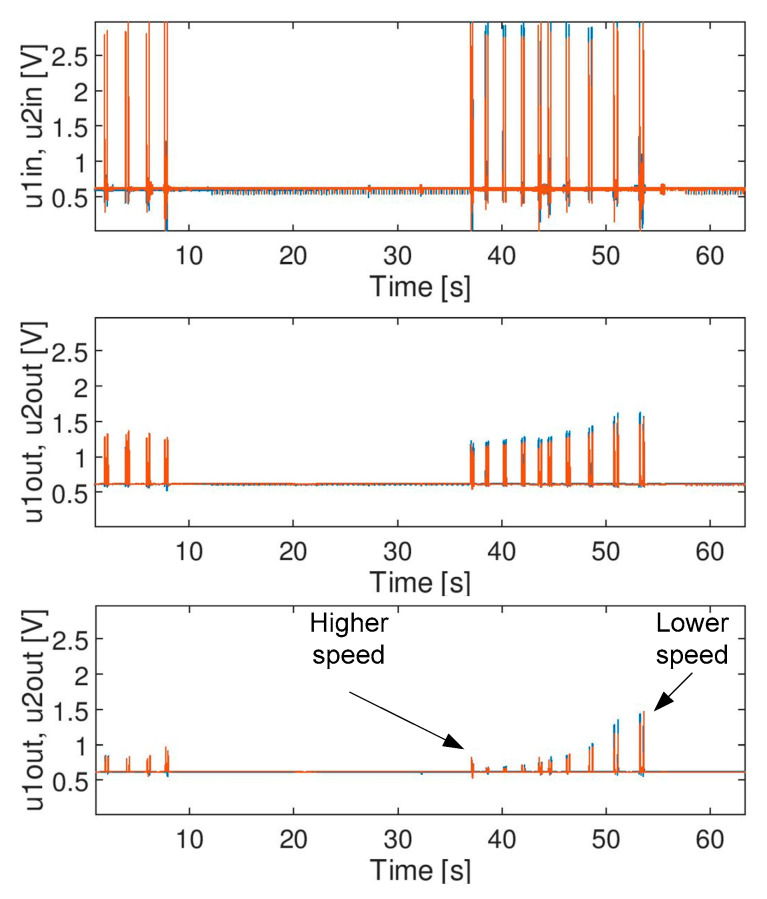
Filtration effect: (**top**)—raw input signal; (**middle**)—MAA; (**bottom**)—MF. Both filters have a window size of 256.

The impact of moving average filter on the signal is not so sudden compared to the impact of a median filter. With respect to selected parameters (i.e., window size), the filter operates correctly at a particular speed of a train, and no risk of malfunction was observed in the case of a set of signals (i.e., a signal does not disappear suddenly when train speed changes so the moving average filter does not cause any hazard in the system). The raw signal (Figure 12 (**top**)) and the effect of filtration for both filters (MAA and MF) for an assumed window size of 256 is presented in Figure 12 (**middle**) and (**bottom**), respectively. It can be seen that the median filter can change (destroy) the signal, if the speed of a train changes. Therefore, the length of the filter buffer for median filter shall be chosen with high safety margins in relation to the maximum permitted train speed (it is defined for each application).

**Figure 13:**
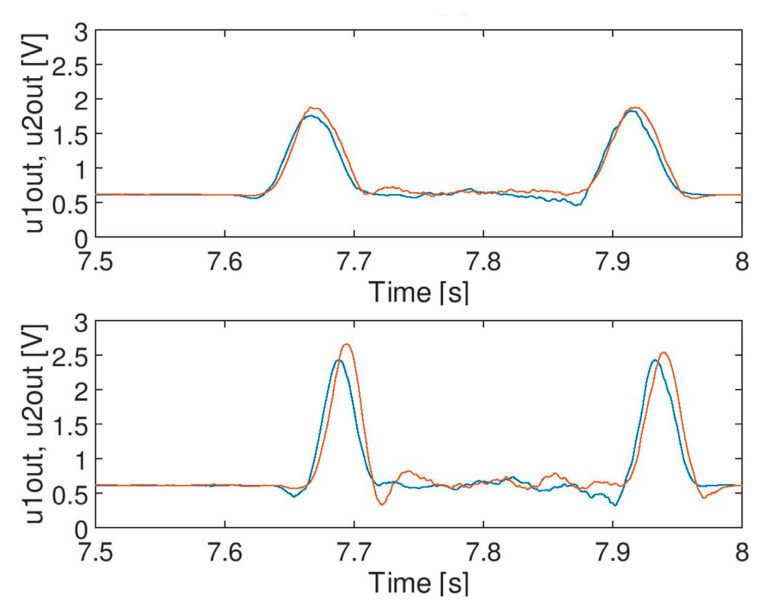
Comparison of filtration of signal C: moving average filter output (**top**) and Savitzky-Golay (polyn. deg. = 3) filter output (**bottom**), both with window size of 127.

**Figure 14:**
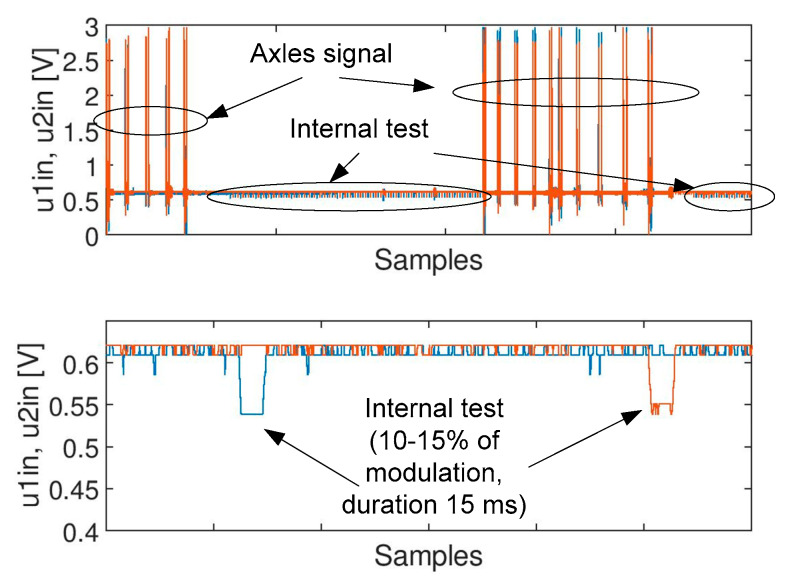
Internal test signal for the wheel detector.

Savitzky-Golay filter allows for implementing an additional filtration parameter (i.e., the degree of polynomial), which can be used to control how the output signal shall follow the input one. Symmetric impulses are effectively removed, and on the other side, the signal is not dumped like in the case of an arithmetic moving average filter (compare Figure 13 (**top**) with Figure 13 (**bottom**)). Moreover, the SG filter is also better for the application, when the signal is modulated due to safety reasons (e.g., a “life test” is performed). Such a modulation is used for checking the correctness of operation of the detector being a subject of the analysis. The “life test” signal (internal test) is shown in Figure 14, where 10–15% amplitude modulation is visible. This change of the signal is also visible in the output signal, otherwise it would cause a failure of the detector.

**Figure 15:**
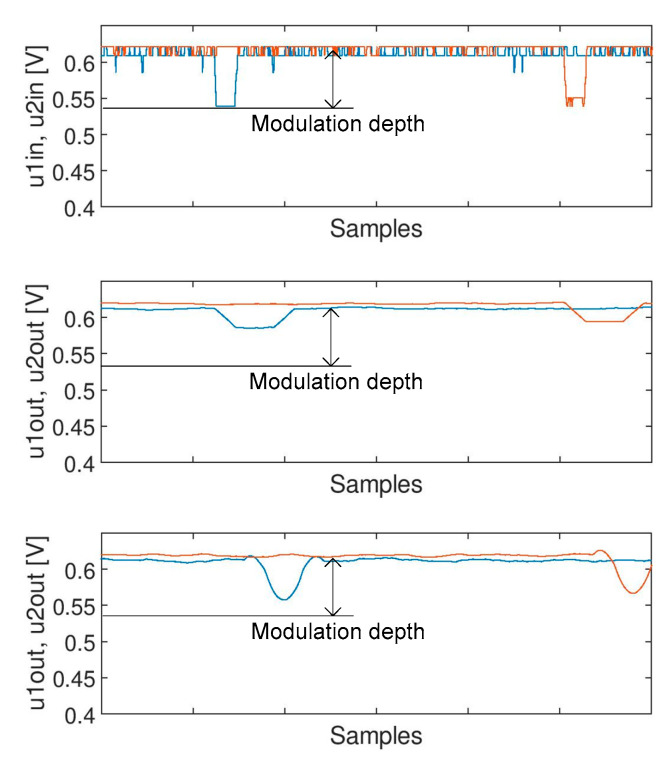
Loss of modulation depth in the output signal.

The loss of the modulation depth in the output signal is shown in Figure 15. The modulation depth is almost lost for moving average filter (it is decreased by 50%), while it can be considered as negligible for Savitzky-Golay filter.

The impact of smoothing filters on the signals can be significant. Each time such a filter is used, its parameters shall be chosen with respect to the maximum speed of the train that is permitted in the application. For a median filter, the safety margin shall be much higher than for moving average filters.

## 7. Conclusions

The appropriate filtration of the wheel detector signals has a positive impact on its proper operation. A correctly configured median filter eliminates impulse noise from the signal (e.g., signal A), but the configuration must consider experience and safety conditions for the system because its impact to the signal for the fast moving wheels must be taken into account. Such a signal may be strongly deformed or completely cut out if the length of the filter (window size) is too big. Research has indicated that MF (window size of 64) eliminates impulses, giving clear signal in the output. Signal B is effectively reduced by MAA and MAS filters (window size of 64). Before filtration, signal B was multiplied by factor 2 to implement the worst case scenario (Figure 4 shows the signal before multiplication). It adds the safety margin and shows that the system can still work correctly and safely (there is no false counting, no errors, and the number of false transitions in the state machine is minimal). Moreover, moving average filter gives the best results with respect to signal C. The output signal is however a bit deformed, but the number of axles is counted correctly for all the thresholds; the Savitzky-Golay filter gives similar results to moving average filter results but in order to achieve the same quality of the signal, the buffer size must be longer. On the other side, the shape of the output signal for axle and test impulse better reflects the input one and thus better reproduces the undisturbed signal. The results of the other filters tested are not better and since their implementation would require more computational resources (power and root calculation), their use is not recommended.

Analysis of the paper filtration methods can be applied to the current installed hardware. Rare but difficult signals can be either eliminated or reduced by the following configuration of filters (as both impulse and continuous disturbances are present in the signal):

A median filter and moving average filter (or even moving average square filter for better signal to noise ratio, however more operations are needed). This setup reduces impulse noise and continuous, but the shape of the signal will be deformed. It can be applied in the systems when the shape itself does not carry out important information. For high speeds, the duration of wheel impulse will be affected after passing through the filters.

A median filter and a polynomial smoothing filter are recommended for cases when a signal carries specific important information in its shape. Such a combination will reproduce the original signal to a greater degree and will maintain the data carried.

Another very important aspect is connected with the implementation of smoothing filters which are not complex and do not consume much of the processing time. Moving average can be calculated in a cumulative way, thereby decreasing the minimum number of operations executed for each new sample to a minimum.

The impact of a filter on a signal shall be taken into consideration with respect to all the filter types. Wrongly configured median filter can completely cut out the signal of wheels, therefore information about the constraints shall be clearly defined in the Safety Related Application Conditions and Application Guidelines for such types of systems. Filter parameters shall be treated as safety critical data and appropriately protected. The filtering functions shall also be implemented and executed in the regime for SIL-4 safety integrity level.

The presented research is an important first stage for evaluation of the method of improving the operation of an axle counter system on a software level. This stage is a kind of preprocessing of the signals which are further analyzed in other software modules.

Moreover, the use of digital filters, that are applied as software modules opens up the possibility of further improvement. It is a possibility of adapting filter parameters so that they could be controlled by a decision module and thereby improve train detection system availability.

## Figures and Tables

**Table 1:** Results gathered for signal A.

Filter Type	Std. Dev. (mV)	Std. Dev. (mV)	(DM) THR = 1.60 V			(DM) THR = 1.20 V			(DM) THR = 0.95 V		
			AXL ^1^	ST1	ST2	AXL ^1^	ST1	ST2	AXL ^1^	ST1	ST2
None	29.48	30.28	1	0	13	1	1	28	1	6	61
MAA (*N_w_* = 32)	17.76	19.61	0	0	3	0	0	4	0	0	6
MAA (*N_w_* = 64)	14.28	15.42	0	0	0	0	0	0	0	0	4
MAG (*N_w_* = 32)	19.90	20.35	0	0	0	0	0	4	0	0	4
MAS (*N_w_* = 32)	19.91	22.60	0	0	4	0	0	6	0	0	8
MAS (*N_w_* = 64)	16.65	19.62	0	0	0	0	0	4	0	0	6
MF (*N_w_* = 32)	16.92	17.28	0	0	0	0	0	4	0	0	4
**MF (*N_w_* = 64)**	**14.76**	**13.01**	**0**	**0**	**0**	**0**	**0**	**0**	**0**	**0**	**0**
SG (31, 3)	22.29	25.13	0	0	4	0	0	8	0	13	10
SG (63, 3)	18.81	20.99	0	0	4	0	0	4	0	0	5

^1^ AXL expected value is zero. MAA: moving average arithmetic filter; MAG: moving average geometric filter; MAS: moving average arithmetic square filter; MF: median filter; SG: Savitzky-Golay; THR: threshold level in (V); AXL: number of counted axles; ST1: status of operation, cumulated number of detected errors. Expected value = 0, ST2: accumulated number of incorrect transitions in the state machine. Value 0 means that there was a clear passage detected (expected). Hence, the lower value → the higher efficiency of filtration.

**Table 2:** Results gathered for signal B, multiplied by 2 and shifted to 0.6 V.

Filter Type	Std. Dev. (mV)	Std. Dev. (mV)	(DM) THR = 1.60 V			(DM) THR = 1.20 V			(DM) THR = 0.95 V		
			AXL ^1^	ST1	ST2	AXL ^1^	ST1	ST2	AXL ^1^	ST1	ST2
None	77.47	46.27	0	0	0	2	15	361	0	1037	4
MAA (*N_w_* = 32)	42.24	30.51	0	0	0	0	0	0	0	0	0
**MAA (*N_w_* = 64)**	**35.75**	**27.36**	**0**	**0**	**0**	**0**	**0**	**0**	**0**	**0**	**0**
MAG (*N_w_* = 32)	45.72	34.64	0	0	0	0	0	0	0	0	0
MAS (*N_w_* = 32)	41.06	29.38	0	0	0	0	0	0	0	0	0
MAS (*N_w_* = 64)	33.28	24.55	0	0	0	0	0	0	0	0	0
MF (*N_w_* = 32)	45.83	33.86	0	0	0	0	0	0	0	0	0
MF (*N_w_* = 64)	39.85	32.91	0	0	0	0	0	0	0	0	0
SG (31, 3)	55.99	39.41	0	0	0	0	0	0	0	0	0
SG (63, 3)	50.75	40.39	0	0	0	0	0	0	0	0	4

^1^ AXL expected value is zero.

**Table 3:** Results gathered for signal C.

Filter Type	Std. Dev. (mV)	Std. Dev. (mV)	(DM) THR = 1.60 V			(DM) THR = 1.20 V			(DM) THR = 0.95 V		
			AXL ^1^	ST1	ST2	AXL ^2^	ST1	ST2	AXL ^2^	ST1	ST2
None	153.46	209.23	−26	1	27	−26	2	25	−27	5	66
**MAA (*N_w_* = 32)**	**72.23**	**83.15**	**−30**	**0**	**0**	**−29**	**0**	**2**	**−29**	**0**	**2**
MAA (*N_w_* = 64)	44.13	61.12	−30	0	0	−29	0	2	−28	0	4
MAG (*N_w_* = 32)	77.96	84.78	−28	2	0	−29	0	2	−28	115	3
MAS (*N_w_* = 32)	72.52	72.14	−30	0	0	−29	0	2	−28	117	3
MAS (*N_w_* = 64)	50.29	46.82	−29	73	1	−28	92	3	−28	104	3
MF (*N_w_* = 32)	71.33	100.16	−29	0	2	−29	1	0	−30	0	0
MF (*N_w_* = 64)	41.46	69.50	−29	0	2	−29	1	0	−30	0	0
SG (31, 3)	107.31	135.78	−28	0	4	−29	0	2	−30	0	3
SG (63, 3)	80.20	88.37	−30	0	0	−29	0	2	−29	0	2

^1^ AXL expected value is 30; ^2^ The AXL expected value is 29.

## References

[1] D’Ariano A. (2009). Innovative Decision Support System for Railway Traffic Control. IEEE Intell. Transp. Syst. Mag..

[2] Hamadache M., Dutta S., Olaby O., Ambur R., Stewart E., Dixon R. (2019). On the Fault Detection and Diagnosis of Railway Switch and Crossing Systems: An Overview. Appl. Sci..

[3] Kokic I.Z., Nikolic M.V., Kosic B.D., Milanovic M.D., Antonic N.M., Stoikovic Z.M. (2014). Railway axle counter remote supervision system. Proceedings of the 2014 22nd Telecommunications Forum Telfor (TELFOR).

[4] Kumar M.M., Murali M.S., Saranya M., Arun S., Jayakrishnan R.P. (2018). A Survey on Crack Detection Technique in Railway Track. Proceedings of the 2018 Conference on Emerging Devices and Smart Systems (ICEDSS).

[5] Yao T., Dai S., Wang P., He Y. (2012). Image based obstacle detection for automatic train supervision. Proceedings of the 2012 5th International Congress on Image and Signal Processing.

[6] Palmer J.W. (2012). The need for train detection. Proceedings of the IET Professional Development Course on Railway Signalling and Control Systems (RSCS 2012).

[7] Garramiola F., Poza J., Madina P., del Olmo J., Ugalde G. (2020). A Hybrid Sensor Fault Diagnosis for Maintenance in Railway Traction Drives. Sensors.

[8] Goundan P.R., Jhunjhunwala A. (1999). Axle counter based block signalling for safe and efficient train operations. Proceedings of the Gateway to 21st Century Communications Village, VTC 1999-Fall, IEEE VTS 50th Vehicular Technology Conference (Cat. No.99CH36324).

[9] Futsuhara K., Mukaidono M. (1989). Realization of a fail-safe train wheel sensor using electromagnetic induction. IEEE Trans. Instrum. Meas..

[10] Yasukawa S., Takagi N., Dong G., Wakao S., Takahashi M., Yagi M., Okutani T. (2015). Design Optimization of Magnetic Sensor for Train Detection. IEEE Trans. Magn..

[11] Han J., He Y., Xiao X., Sheng X., Zhao G., Jin X. (2017). Effect of Control Measures on Wheel/Rail Noise When the Vehicle Curves. Appl. Sci..

[12] Wang J., Wu K., Sim A., Hwangbo S. (2017). Convolutional Filtering for Accurate Signal Timing from Noisy Streaming Data. Proceedings of the 2017 IEEE 15th Intl Conf on Dependable, Autonomic and Secure Computing, 15th Intl Conf on Pervasive Intelligence and Computing, 3rd Intl Conf on Big Data Intelligence and Computing and Cyber Science and Technology Congress(DASC/PiCom/DataCom/CyberSciTech).

[13] He J., Sun C., Wang P. (2019). Noise Reduction for MEMS Gyroscope Signal: A Novel Method Combining ACMP with Adaptive Multiscale SG Filter Based on AMA. Sensors.

[14] Jiang Q., Wu W., Jiang M., Li Y. (2017). A New Filtering and Smoothing Algorithm for Railway Track Surveying Based on Landmark and IMU/Odometer. Sensors.

[15] European Committee for Electrotechnical Standardization (CENELEC) (2011). CENELEC Railway Applications. Communication, Signaling and Processing Systems. Software for Railway Control and Protection Systems.

[16] Garramiola F., Poza J., Madina P., del Olmo J., Almandoz G. (2018). A Review in Fault Diagnosis and Health Assessment for Railway Traction Drives. Appl. Sci..

[17] Tadeusiewicz M., Hałgas S. (2017). A Systematic Method for Arranging Diagnostic Tests in Linear Analog DC and AC Circuits. J. Electron. Test..

[18] Stratigopoulos H.-G. (2018). Machine learning applications in IC testing. Proceedings of the 2018 IEEE 23rd European Test Symposium (ETS).

[19] Li Z., Wang Y., Wang K.-S. (2017). Intelligent predictive maintenance for fault diagnosis and prognosis in machine centers: Industry 4.0 scenario. Adv. Manuf..

[20] Grzechca D.E. (2015). Construction of an Expert System Based on Fuzzy Logic for Diagnosis of Analog Electronic Circuits. Int. J. Electron. Telecommun..

[21] Grzechca D. (2011). Soft Fault Clustering in Analog Electronic Circuits with the Use of Self Organizing Neural Network. Metrol. Meas. Syst..

[22] European Committee for Electrotechnical Standardization (CENELEC) (2003). CENECEC Railway Applications. Communication, Signaling and Processing Systems. Safety Related Electronics Systems for Singalling.

[23] European Committee for Electrotechnical Standardization (CENELEC) (2003). CENELEC Railway Applications. Compatibility between Rolling Stock and Train Detection Systems.

[24] Zamani A., Mirabadi A. (2011). Analysis of Sensor Orientation in Railway Axle Counters Using Response Surface Methodology.

[25] Brockmann E.M., Kwan B.W., Tung L.J. (1997). Audio detection of moving vehicles. Proceedings of the 1997 IEEE International Conference on Systems, Man, and Cybernetics, Computational Cybernetics and Simulation.

[26] Wei C.L., Lai C.C., Liu S.-Y., Chung W.H., Ho T.K., Tam H.-Y., Ho S.L., McCusker A., Kam J., Lee K.Y. (2010). A Fiber Bragg Grating Sensor System for Train Axle Counting. IEEE Sens. J..

[27] Ho T.K., Liu S.Y., Ho Y.T., Ho K.H., Wong K.K., Lee K.Y., Tarn H.Y., Ho S.L. (2008). Signature analysis on wheel-rail interaction for rail defect detection. Proceedings of the 4th IET International Conference on Railway Condition Monitoring (RCM 2008).

[28] Smith S.W. (1997). The Scientist & Engineer’s Guide to Digital Signal Processing.

[29] Thampi S.M., Bandyopadhyay S., Krishnan S., Li K.-C., Mosin S., Ma M. (2015). Advances in Signal Processing and Intelligent Recognition Systems. Proceedings of the Second International Symposium on Signal Processing and Intelligent Recognition Systems (SIRS-2015).

[30] Dercosi Persichini R., Di Febo D., Cala V., Malta C., Orlandi A. (2015). EMC Analysis of Axle Counters in the Italian Railway Network. IEEE Trans. Electromagn. Compat..

[31] Guiñón J., Ortega E., García-Antón J., Pérez-Herranz V. Moving average and Savitzki-Golay smoothing filters using Mathcad. Proceedings of the International Conference on Engineering Education—ICEE 2007.

[32] Press W.H., Teukolsky S.A. (1990). Savitzky-Golay Smoothing Filters. Comput. Phys..

[33] Nahiyan K.M.T., Amin A.A. (2017). Removal of ECG Baseline Wander using Savitzky-Golay Filter Based Method. Bangladesh J. Med. Phys..

[34] Kawala-Sterniuk A., Podpora M., Pelc M., Blaszczyszyn M., Gorzelanczyk E.J., Martinek R., Ozana S. (2020). Comparison of Smoothing Filters in Analysis of EEG Data for the Medical Diagnostics Purposes. Sensors.

